# Value of 3D‐High Resolution Anorectal Manometry Before and After Fistulotomy for Simple Perianal Fistulas: A Prospective Cohort Study

**DOI:** 10.1111/ans.70845

**Published:** 2026-07-08

**Authors:** Justin Y. van Oostendorp, Pieter van Hagen, Ruben Veldkamp, Lisette Dekker, Ingrid J. M. Han‐Geurts

**Affiliations:** ^1^ Department of Surgery Amsterdam UMC Amsterdam the Netherlands; ^2^ Department of Surgery Proctos Kliniek Bilthoven the Netherlands

**Keywords:** anorectal manometry, fecal incontinence, fistulotomy, perianal fistula, quality of life

## Abstract

**Aim:**

Surgical treatment of perianal fistulas is challenging due to the risk of postoperative fecal incontinence, which can significantly impair quality of life. Fistulotomy is commonly used as an effective treatment for simple fistulas with limited sphincter involvement, assuming a minimal risk of continence deterioration. However, partial sphincter division may still affect anorectal pressures. This study prospectively evaluates changes in anal sphincter function using three‐dimensional high resolution anorectal manometry (3D‐HRAM) alongside continence and quality of life outcomes before and after fistulotomy.

**Methods:**

A prospective single‐center cohort study was conducted at a tertiary referral center in the Netherlands. Adults undergoing fistulotomy for simple perianal fistulas were included. Pre‐ and postoperative assessments included clinical evaluation, 3D‐endoanal ultrasound, 3D‐HRAM, and functional outcome measures for fecal incontinence and quality of life. Long‐term outcomes were assessed via standardized questionnaires at 12 months postoperatively. Primary outcomes were changes in anorectal pressures, continence deterioration, and quality of life.

**Results:**

Among 47 patients, 26% experienced continence deterioration despite unchanged mean sphincter pressures postoperatively. This subgroup showed lower preoperative fistula tract pressures and altered voluntary squeeze pressures on 3D‐HRAM. Despite this, overall quality of life improved significantly following fistula healing.

**Conclusion:**

3D‐HRAM allows for regional pressure mapping in perianal fistula but does not reliably predict continence deterioration after fistulotomy. Given the low risk of severe incontinence, routine preoperative 3D‐HRAM is not warranted for simple fistulas. Future research should refine continence assessment and clarify its role in complex fistulas, where the risk impairment is higher.

**Trial Registration:** Dutch trial register: NL78713.018.21

## Graphical abstract

In this prospective cohort study, 3D‐high resolution anorectal manometry provided detailed regional pressure assessment but failed to predict postoperative continence deterioration after fistulotomy for simple perianal fistulas. These findings highlight the need for a continence assessment tool that better distinguishes true fecal incontinence from fistula‐related leakage.

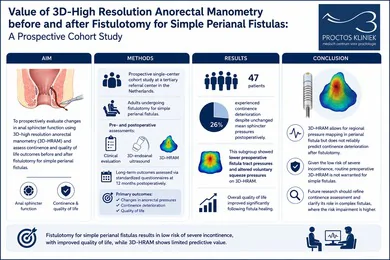

## 1 Introduction

The surgical management of perianal fistulas is challenging, as treatment must balance fistula healing with the preservation of continence. Postoperative fecal incontinence (FI) is a serious complication that can significantly reduce quality of life (QoL) [1, 2]. It often leads to activity restrictions, lifestyle limitations, emotional distress, and social embarrassment [3, 4]. Given that perianal fistula is a benign condition, both patients and surgeons face the dilemma of selecting an intervention that effectively cures the disease without compromising function. Since the extent of sphincter involvement is a key determinant of continence outcomes, thorough preoperative evaluation and individualized treatment planning are essential [3, 5].

Fistulotomy is the preferred treatment for simple fistulas (intersphincteric and low trans‐sphincteric), offering high healing rates [6, 7, 8]. However, postoperative FI rates vary widely (4%–62%), emphasizing the need for careful patient selection and a better understanding of factors influencing functional outcomes [9, 10, 11, 12, 13, 14, 15, 16]. While sphincter‐sparing techniques are generally favored for complex or high fistulas to minimize incontinence risk, some studies suggest that fistulotomy can also be safely performed in selected cases within this category [7, 15, 17]. Nonetheless, recent data indicate that even partial sphincter division (up to 50%) can result in mild FI in up to 31% of patients [16, 18]. These reported rates vary due to differences in definitions, assessment tools, and follow‐up durations.

Advances in imaging and functional assessment, such as three‐dimensional endoanal ultrasound (3D‐EUS) and anorectal manometry (ARM), have improved our ability to evaluate perianal fistulas and predict surgical outcomes [18, 19, 20, 21]. These tools allow for detailed anatomical mapping and pressure profiling, offering valuable insights into sphincter function, which is closely linked to postoperative continence [13, 22, 23, 24, 25].

While traditional ARM provides general (mean) pressure measurements, it lacks the resolution to detect localized variations within the anal canal. Three‐dimensional high resolution anorectal manometry (3D‐HRAM) surmounts this limitation by enabling regional pressure mapping and potentially detecting subtle dysfunctions [26]. However, despite these advancements, prospective data linking 3D‐HRAM findings to postoperative continence and QoL remain scarce, particularly for simple perianal fistulas, where fistulotomy remains the standard but not entirely risk‐free treatment of choice.

The aim of this study was to evaluate pre‐ and postoperative anorectal pressure profiles using 3D‐HRAM in patients undergoing fistulotomy for simple perianal fistula, and to explore the relationship between these profiles, their postoperative changes, FI symptoms, and related quality of life.

## 2 Methods

### 2.1 Study Design, Setting, and Population

This prospective single‐center cohort study was conducted at a tertiary referral center in the Netherlands and adhered to the STROBE guidelines. The study was approved by the Amsterdam UMC Medical Ethics Committee (MEC no. 2021_188) and was conducted in accordance with the Declaration of Helsinki. Written informed consent was obtained from all participants prior to enrollment. The trial was prospectively registered in the Dutch Trial Register (NL78713.018.21).

Adults aged 18–80 years undergoing lay‐open fistulotomy for simple perianal fistulas (intersphincteric or trans‐sphincteric with ≤ 1/3 external sphincter involvement) were eligible. Diagnosis was confirmed by clinical examination and 3D‐EUS. The classification of fistulas and the decision that fistulotomy was technically feasible and appropriate were established during a consensus meeting with multiple colorectal surgeons from our clinic, based on clinical history, physical examination, and 3D‐EUS findings. Patients were followed for 12 months.

Exclusion criteria included complex fistulas (multiple external openings, high trans‐sphincteric, supra‐, or extra‐sphincteric), non‐cryptoglandular etiology (e.g., Crohn's disease), and prior pelvic radiation.

### 2.2 Study Outcomes

Demographics, medical history (smoking status, diabetes mellitus, obstetric history, and prior anorectal diseases), and fistula characteristics (previous treatments, tract anatomy, internal fistula opening height, and external sphincter involvement) were recorded.

The primary outcome was fecal continence deterioration, defined as a composite endpoint: (1) any increase in Wexner score postoperatively, regardless of baseline score, or (2) a postoperative Wexner score > 4, even if improved from baseline [27]. This threshold was based on expert consensus in the absence of validated cutoff values. Given the hypothesized role of sphincter function in continence outcomes, we examined the association between continence deterioration and pre/postoperative anal pressure profiles using 3D‐HRAM, with particular focus on regional pressure differences over the fistula tract.

Secondary outcomes included FI‐related quality of life (FIQL), impact on daily activities, patient‐reported acceptability, sphincter defects on ultrasound, and recurrence rate.

### 2.3 Data Collection and Measurements

Data was collected through clinical assessments and electronic questionnaires. Baseline assessment included digital rectal examination, 3D‐EUS, and 3D‐HRAM. Follow‐up evaluations occurred at 4 and 12 months.

### 2.4 Three‐Dimensional Endoanal Ultrasonography

3D‐EUS (Hawk type 2050, B‐K Medical, Denmark) was performed pre‐ and postoperatively after injection of 1 cc 2% hydrogen peroxide into the fistula to enhance fistula tract visualization. Patients were examined in the left lateral position by an experienced proctologic surgeon [26]. Fistulas were classified using the Parks system [28]. Sphincter integrity was assessed by measuring internal fistula opening height, total internal sphincter length, and the presence of sphincter defects. The proportion of muscle division was quantified postoperatively by measuring the length of the sphincter defect. All images were reviewed independently by three investigators who were masked to the study outcomes, with discrepancies resolved by consensus.

### 2.5 Three‐Dimensional High Resolution Anorectal Manometry

3D‐HRAM was performed using a 256‐sensor probe (Given Imaging, Duluth, GA, USA) preoperatively and at 4 months postoperatively. The 4‐month time point was chosen to reflect early functional changes after fistulotomy once initial wound healing had occurred. Measurements followed standardized protocols with patients in the left lateral position [26, 29]. Pressure data was analyzed using ManoView software (Given Imaging, Duluth, GA, USA) and presented in both 2D and 3D formats [26, 29]. Anal sphincter pressure in selected areas was measured using the SmartMouse technology, with repeated measurements averaged for reliability.

Primary manometric parameters included mean resting pressure (RPmean), maximal squeeze pressure (SPmax), and fistula tract pressure (FTP). The fistula tract was visually identified on the 3D pressure topography as a localized area of low pressure or pressure drop, represented by a color pattern that clearly differed from the surrounding tissue. FTP was defined as the pressure measured locally within the fistula tract at the level of the internal opening using the SmartMouse function. Absolute (mmHg) and relative (%) pressure drops were calculated as indicators of localized dysfunction. Reference values were drawn from published norms stratified by age and gender [30, 31, 32].

### 2.6 Questionnaires

Fecal incontinence was assessed using the Cleveland Clinical Florida Incontinence Score (CCFIS; also referred to as the Wexner score, 0–20) [27]. Quality of life was measured using the Fecal Incontinence Quality of Life (FIQL) scale (0–16, across four domains) [4]. Additional patient‐reported outcomes included the ProctoPROM (0–10) and acceptability of their condition (yes/no) [33].

### 2.7 Surgical Procedure

Fistulotomy was performed under general anesthesia in lithotomy position. The fistula tract was laid open after intraoperative assessment of the extent of sphincter involvement prior to division. Postoperative care included routine wound management, analgesia, and scheduled follow‐up.

### 2.8 Statistical Analyses

Statistical analyses were performed using SPSS (version 28, IBM, Armonk, NY, USA). Quantitative data were expressed as medians with interquartile ranges (IQR), and categorical variables as frequencies with percentages. Continuous variables were compared using Wilcoxon signed‐rank tests, while categorical data were analyzed using Fisher's exact test. Changes in anorectal pressures, Wexner scores, and FIQL scores over time were analyzed both individually and at the group level, with statistical significance set at *p* < 0.05. Subgroup analyses were conducted for patients with continence deterioration. Sphincter involvement was categorized based on the extent of sphincter division (< 33%, 33%–66%, > 66%). Uni‐ and multivariate regression analyses were performed to identify predictors of postoperative fecal continence deterioration.

## 3 Results

### 3.1 Baseline Characteristics

Between January 2022 and December 2024, 122 patients met the eligibility criteria, of whom 59 consented and underwent baseline 3D‐HRAM. In 12 cases, fistulotomy was not performed due to various reasons (Figure 1), leaving 47 patients in the final study cohort (Table 1). Postoperative follow‐up was completed in 45 patients, as two did not undergo postoperative 3D‐HRAM: one declined after symptom relief, and one due to travel difficulties. Postoperative questionnaires were completed by all 47 patients, with a completion rate of 94%.

**FIGURE 1:**
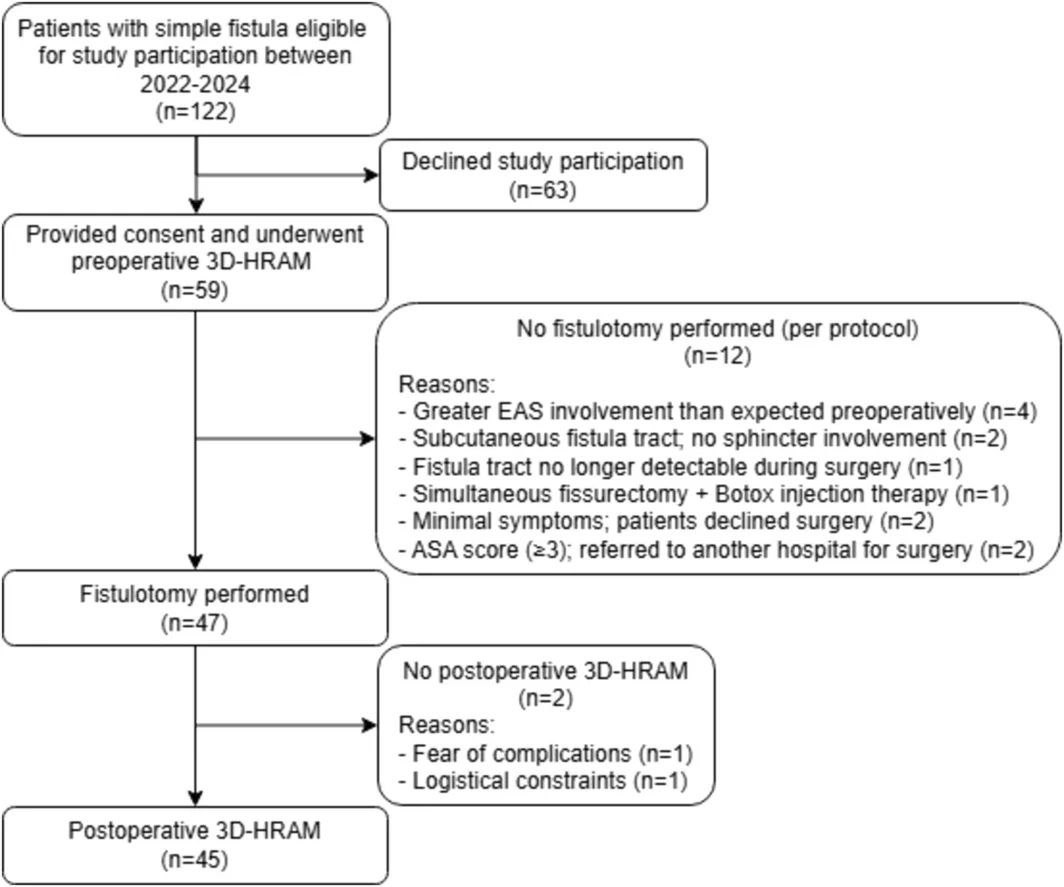
Study flowchart.

**TABLE 1:** Baseline characteristics.

Baseline characteristics	Total cohort (*n* = 47)
Age (years), mean (SD)	44 (10.3)
Sex ratio (M:F)	32:15
BMI (kg/m^2^), mean (SD)	25.9 (3.5)
ASA‐classification (I:II)	40:7
Smoking, *n* (%)	5 (11)
History of vaginal delivery, *n*/*N* (%)	8/15 (53)
Number of vaginal deliveries (1:2:3)	1:2:5
Perineal tear during delivery	2 (25)
Episiotomy performed	6 (75)
History of fissura ani, *n* (%)	15 (32)
Tertiary referrals, *n* (%)	27 (57)
History of abces/seton drainage, *n* (%)	18 (38)
History of fistula closure attempts, *n* (%)	5 (11)
Advancement flap (AF)	1 (2)
Ligation of the intershpincteric fistula tract (LIFT)	1 (2)
Fistulotomy	3 (6)
Seton present at time of fistulotomy, *n* (%)	6 (13)
Duration of seton (months), mean (SD)	5 (2.8)

3D‐EUS characteristics	
Type of tract (Parks), *n* (%)	
Intersphincteric	19 (40)
Trans‐sphincteric	28 (60)
Location of IFO, *n* (%)	
Posterior	23 (49)
Anterior	21 (45)
Lateral	3 (6)
IFO height (mm), mean (SD)	8 (4)
IAS total length (mm), mean (SD)	23 (7)
IFO/IAS % height, mean (SD)	36 (16)
< 33%, *n* (%)	19 (40)
33%–66%, *n* (%)	28 (56)
> 66%, *n* (%)	2 (4)
Defect internal anal sphincter, *n* (%)	4 (8)
Defect external anal sphincter, *n* (%)	2 (4)

Abbreviations: ASA, American Society of Anesthesiologists; BMI, body mass index; IAS, internal anal sphincter; IFO, internal fistula opening.

The mean age was 44 years (standard deviation (SD) 10.3), and 68% (*n* = 32) were male. Among female patients, 53% had a history of vaginal delivery with reported obstetric injury. Over half (57%) were tertiary referrals, including 38% with prior abscess or seton drainage and 11% with previous failed fistula closure attempts. A history of anal fissure was reported in 32%, and 11% had received Botox treatment. At surgery, 13% had a seton in place (mean duration 5 months, SD 2.8). Fistulas were intersphincteric in 40% and trans‐sphincteric in 60%. External fistula openings were located anteriorly in 30% of fistulas, posteriorly in 43%, and laterally in 23%. Median follow‐up was 22 months (range 12–28).

### 3.2 Primary Outcomes

The median Wexner score at baseline was 4 (IQR 1 − 7), decreasing significantly to 1 at 4 months postoperatively (IQR 0–3, *p* = 0.022) and remaining stable at 12 months postoperatively (IQR 0–2, *p* < 0.001) (Figure 2A). At baseline, 21% of patients reported full continence (Wexner score 0). Another 34% of patients reported a Wexner score of 1–4, 30% a score of 5–8, and 11% a score of 9–20 at baseline. Baseline questionnaires were missing for two patients (4%).

**FIGURE 2:**
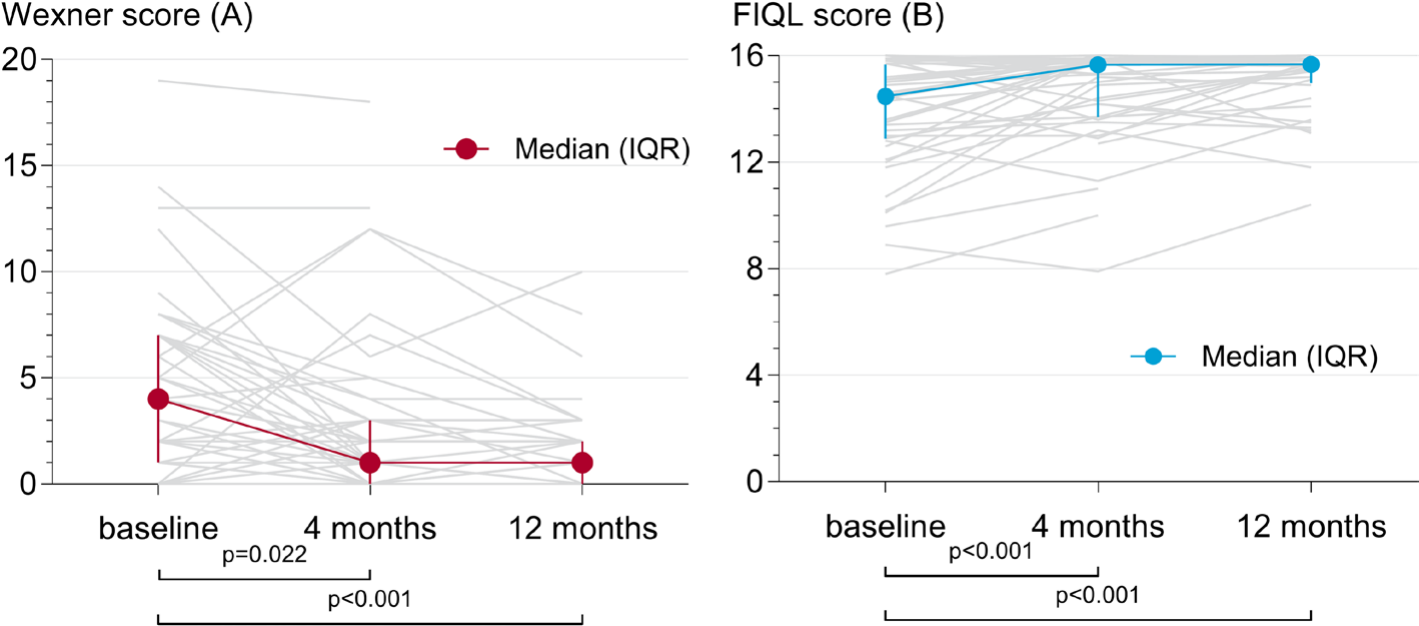
Total cohort. (A) Wexner scores, (B) FIQL scores.

Regarding fecal continence deterioration, defined as either worsening of Wexner score (*n* = 9) or a postoperative Wexner score > 4 (*n* = 5), a total of 12 patients (26%) met at least one of these criteria, with two patients fulfilling both. No significant baseline differences were observed between patients with and without deterioration (Table 4). Subcategory breakdowns of Wexner scores over time are presented in Table 2.

**TABLE 2:** Wexner score subcategories during follow‐up.

Categories > 0 frequency (score)	Baseline, *n*/*N* (%)	4‐months, *n*/*N* (%)	12‐months, *n*/*N* (%)	*p* *
Gas	31/45 (69)	29/47 (62)	25/41 (63.4)	0.616
< 1×/month (1)	12 (27)	13 (28)	15 (36.5)	
< 1×/week (2)	9 (20)	7 (15)	7 (17.1)	
< 1×/day (3)	5 (11)	6 (13)	3 (7.3)	
> 1×/day (4)	5 (11)	3 (6)	1 (2.4)	
Liquid	22/45 (49)	17/47 (36)	10/41 (24)	0.004
< 1×/month (1)	11 (24)	10 (21)	8 (20)	
< 1×/week (2)	6 (13)	4 (9)	1 (2)	
< 1×/day (3)	2 (4)	1 (2)	0 (0)	
> 1×/day (4)	3 (7)	2 (4)	1 (2)	
Solid	8/45 (17)	6/47 (12)	3/41 (7)	0.003
< 1×/month (1)	5 (11)	2 (4)	1 (2.4)	
< 1×/week (2)	0 (0)	1 (2)	1 (2.4)	
< 1×/day (3)	2 (4)	3 (6)	0 (0)	
> 1×/day (4)	1 (2)	0 (0)	1 (2.4)	
Pad usage	14/45 (31)	5/47 (11)	2/41 (5)	0.006
< 1×/month (1)	3 (7)	2 (4)	1 (2.4)	
< 1×/week (2)	2 (4)	0 (0)	1 (2.4)	
< 1×/day (3)	1 (2)	1 (2)	0 (0)	
> 1×/day (4)	8 (18)	2 (4)	0 (0)	
Impact lifestyle	16/45 (36)	7/47 (15)	2/41 (5)	0.004
< 1×/month (1)	4 (9)	1 (2)	0 (0)	
< 1×/week (2)	7 (16)	1 (2)	1 (2.4)	
< 1×/day (3)	3 (7)	2 (4)	0 (0)	
> 1×/day (4)	2 (4)	3 (6)	1 (2.4)	
Total score^a^	4 (0–19)	1 (0–18)	1 (0–10)	< 0.001**

^a^ Median (range).

* Last available score compared to baseline.

** Wilcoxon signed‐rank test.

### 3.3 Manometry Results

Preoperatively, RPmean was normal (> 50 mmHg) in all patients (100%), and SPmax was normal (> 85 mmHg) in 46/47 patients (98%). Postoperatively, both remained largely stable though RPmean decreased slightly but significantly (−6 mmHg, *p* = 0.029). Baseline RPmean and SPmax showed no correlation with postoperative Wexner scores (Figure 3A,B), nor did their postoperative changes (Figure 3C,D).

**FIGURE 3:**
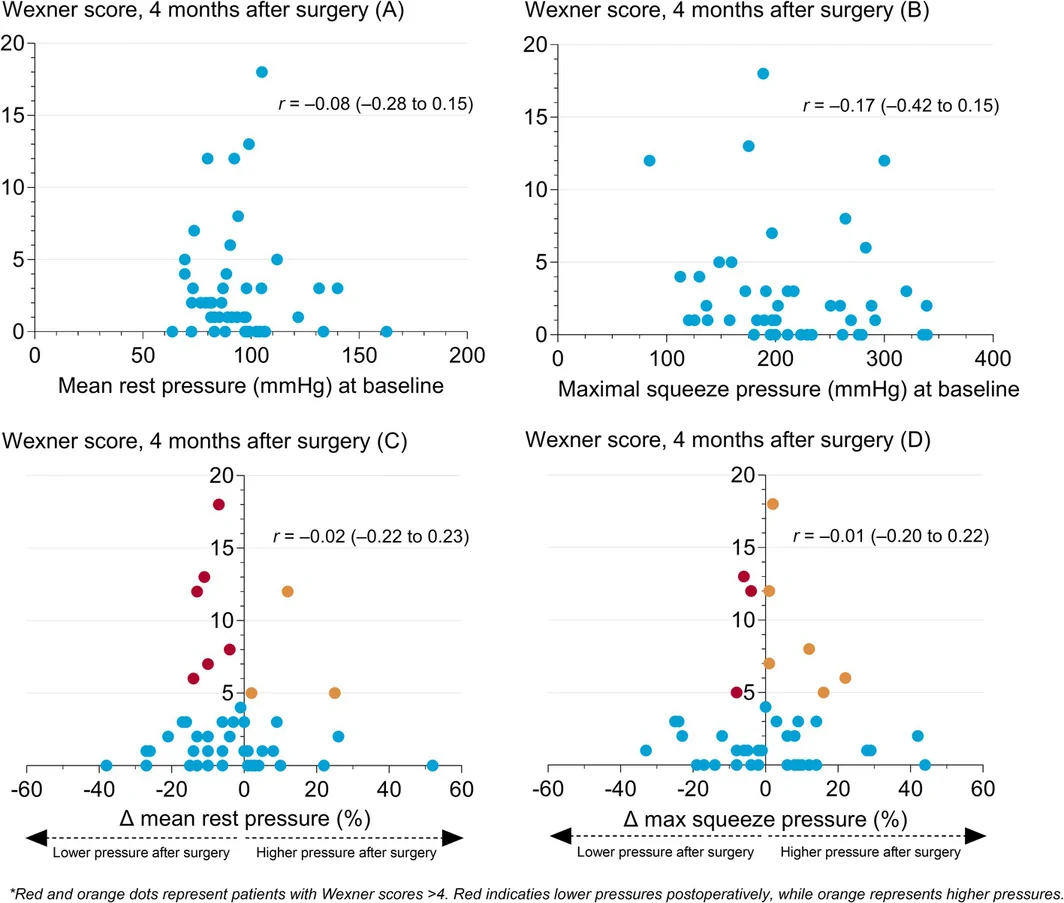
Relationship between RPmean and SPmax with Wexner score at baseline (A and B) and postoperatively (C and D).

### 3.4 Fistula Tract Pressures

Fistula tracts were visually identifiable on 3D‐HRAM in ~80% of patients, both at rest and during maximal voluntary squeeze. Visibility was slightly lower postoperatively. In patients with identifiable tracts, FTPs were consistently lower than RPmean and SPmax, both pre‐ and postoperatively (Table 3).

**TABLE 3:** 3D‐HRAM measurements at baseline and 4 months after fistulotomy.

Parameters	Total cohort (*n* = 47)				Subgroup with fecal continence deterioration (*n* = 12)			
	Baseline	Postoperative	Difference	*p* *	Baseline	Postoperative	Difference	*p* *
RPmean^a^	92 (82–104)	88 (73–102)	–6 (−12 to +3)	0.027	89 (74–98)	81 (68–95)	–8 (−11 to 0)	0.050
SPmax^a^	202 (175–270)	215 (165–280)	+3 (−14 to +22)	0.400	204 (178–278)	209 (165–286)	+4 (−9 to +23)	0.480
FTP―rest^a^	61 (43–73)	44 (36–60)	–7 (−27 to 0)	0.001	43 (36–64)	39 (30–49)	–7 (−3 to −15)	0.016
FTP―squeeze^a^	109 (87–147)	82 (68–112)	–18 (−41 to +5)	0.005	103 (66–184)	97 (68–118)	−17 (−3 to −41)	0.075
Pressure drop―rest^a^	31 (11–56)	43 (30–56)	−12 (−21 to +8)	0.017	40 (8–60)	41 (30–57)	−3 (−14 to +7)	0.213
Pressure drop―squeeze^a^	99 (70–152)	129 (72–183)	−19 (−43 to +1)	0.003	71 (25–147)	108 (52–185)	−19 (−39 to 0)	0.016
Pressure drop (%)―rest	35 (13–55)	50 (32–61)	+12 (−3 to +23)	0.002	51 (12–58)	55 (37–62)	+8 (−1 to +18)	0.037
Pressure drop (%)―squeeze	50 (36–58)	60 (44–71)	+9 (−4 to +20)	0.015	34 (22–64)	48 (42–73)	+9 (+2 to +20)	0.026

*Note:* All values are medians with corresponding interquartile ranges (IQR).

^a^ Units: mmHg.

* Wilcoxon signed‐rank tests.

Preoperatively, the median absolute pressure drop over the fistula tract was 31 mmHg, with 53% of patients (20/38) exhibiting a pressure drop > 30 mmHg. However, this was not associated with fecal continence deterioration. Postoperatively, pressures at the former tract site declined further (44 vs. 61 mmHg; *p* = 0.001). Additionally, absolute pressure drops became more pronounced during both rest (*p* = 0.017) and squeeze (*p* = 0.003), while relative pressure drops increased by 12% at rest (*p* = 0.002) and 9% during squeeze (*p* = 0.015).

Due to variability in pressure changes, a subgroup analysis was performed in patients with continence deterioration (*n* = 12) to assess whether distinct pressure patterns were present.

### 3.5 Subgroup Analysis: Continence Deterioration

In the subgroup of patients with fecal continence deterioration (*n* = 12), fistula tract pressures and pressure drops were analyzed separately (Table 3 right side).

Similar to the total cohort, all 12 patients had normal RPmean preoperatively, while SPmax was normal in 92% (11/12). Postoperative pressure changes were minimal (RPmean −8 mmHg, *p* = 0.050; SPmax +4 mmHg, *p* = 0.480).

Preoperatively, fistula tract pressures at rest were lower in this subgroup compared to the total cohort (43 vs. 61 mmHg). Postoperatively, these pressures decreased further to median 39 mmHg (*p* = 0.016), with significant increases in relative pressure drops postoperatively at rest (*p* = 0.037) and during maximal squeeze (*p* = 0.026), suggesting greater localized pressure reduction within the anal canal of affected patients.

During maximal squeeze, the median preoperative pressure drop was smaller compared to the total cohort (71 vs. 99 mmHg). Postoperatively, this drop increased further (median 19 mmHg, *p* = 0.016), but remained less pronounced than in the overall cohort. Similarly, relative pressure drops were lower both preoperatively (34% vs. 50%) and postoperatively (48% vs. 60%) in this subgroup, indicating a postoperative decline in contractile force at the fistula site, though to a lesser extent than in the total cohort.

Regression analysis identified lower preoperative FTP (OR 0.92, 95% CI 0.86–0.98; *p* = 0.015) and smaller relative pressure drop (%) during maximal squeeze (OR 0.92, 95% CI 0.87–0.99; *p* = 0.019) as independent predictors of postoperative fecal continence deterioration (Table 4). Lower FTP was associated with higher odds of continence deterioration (1 mmHg lower preoperative FTP: OR 1.09, 95% CI 1.02–1.16), while greater relative pressure drops during squeeze appeared to have a protective effect. No associations were found with age, gender, obstetric history, or prior surgery.

**TABLE 4:** Logistic regression analysis for fecal continence deterioration after fistulotomy.

Variables	*n* (%)	Univariable analysis		Multivariable analysis	
		OR (95% CI)*	*p* *	OR (95% CI)*	*p* *
Age^a^	44 (10)	0.97 (0.90–1.04)	0.334		
Gender					
Male	32 (68)	[Referent]			
Female	15 (32)	1.79 (0.46–6.97)	0.404		
BMI^a^	26 (3.5)	0.99 (0.82–1.19)	0.907		
Smoking					
No	42 (89)	[Referent]			
Yes	5 (11)	2.13 (0.31–14.62)	0.440		
Vaginal deliveries					
No	39 (83)	[Referent]			
Yes	8 (17)	2.00 (0.40–10.04)	0.400		
Prior fistula repair attempts					
No	42 (89)	[Referent]			
Yes	5 (11)	0.70 (0.07–7.00)	0.765		
Prior fistula surgery (incl. drainage/seton)					
No	27 (57)	[Referent]			
Yes	20 (43)	0.95 (0.25–3.60)	0.943		
Baseline Wexner score, median (IQR)	4 (1–7)	1.13 (0.97–1.32)	0.113	1.10 (0.87–1.39)	0.416
Fistula type					
Intersphincteric	19 (40)	[Referent]			
Trans‐sphincteric	28 (60)	0.59 (0.16–2.22)	0.436		
Location IFO					
Posterior	22 (47)	[Referent]			
Anterior	22 (47)	0.44 (0.11–1.77)	0.247		
Lateral	3 (6)	—			
IFO height (mm)^a^	8 (3.7)	0.87 (0.71–1.05)	0.154	0.89 (0.68–1.15)	0.367
IFO/IAS height^a^	36 (16)	0.99 (0.95–1.03)	0.670		
Fistula height IFO/IAS (%)					
< 33%	19 (40)	[Referent]			
33%–66%	26 (56)	0.52 (0.13–2.04)	0.345		
> 66%	2 (4)	2.17 (0.11–40.8)	0.606		
Baseline IAS defect					
No	43 (91)	[Referent]			
Yes	4 (9)	3.44 (0.42–28.01)	0.247		
Baseline RPmean^a^	94 (19)	0.98 (0.94–1.02)	0.291		
Baseline SPmax^a^	202 (65)	0.99 (0.98–1.00)	0.617		
Baseline FTP—rest^a^	60 (21)	0.96 (0.92–1.00)	0.061	0.92 (0.86–0.98)	0.015
Baseline FTP—squeeze^a^	114 (40)	1.00 (0.99–1.02)	0.663		
Baseline pressure drop—rest^a^	34 (27)	1.01 (0.98–1.04)	0.457		
Baseline relative pressure drop (%)—rest^a^	34 (24)	1.02 (0.99–1.05)	0.210		
Baseline pressure drop—squeeze^a^	109 (56)	0.99 (0.98–1.00)	0.310		
Baseline relative pressure drop (%)—squeeze^a^	47 (17)	0.97 (0.93–1.01)	0.154	0.92 (0.87 to 0.99)	0.019

Abbreviations: BMI, body mass index; CI, confidence interval; FTP, fistula tract pressure; IFO, internal fistula opening. RPmean, mean resting pressure; SPmax, maximal squeeze pressure.

* Variables with *p* < 0.20 in the univariate regression model were included in the multivariate regression model, where *p* < 0.05 was considered statistically significant.

^a^ Continuous variables are presented as means (standard deviation).

### 3.6 Secondary Outcomes

Quality of life, assessed by the FIQL scale, significantly improved postoperatively. Median FIQL scores increased from 14.5 (IQR 12.9–15.7) at baseline to 15.7 (IQR 13.7–15.8, *p* < 0.001) at 4 months, and remained stable at 15.7 (IQR 15.0–15.9, *p* < 0.001) at 12 months (Figure 2B). All FIQL domains demonstrated postoperative improvement, with the depression/self‐perception subscale showing the greatest variation among patients (Figure 4). Patients with fecal incontinence deterioration reported lower quality of life across all four FIQL domains postoperatively, but their scores became comparable to the rest of the cohort at 1 year.

**FIGURE 4:**
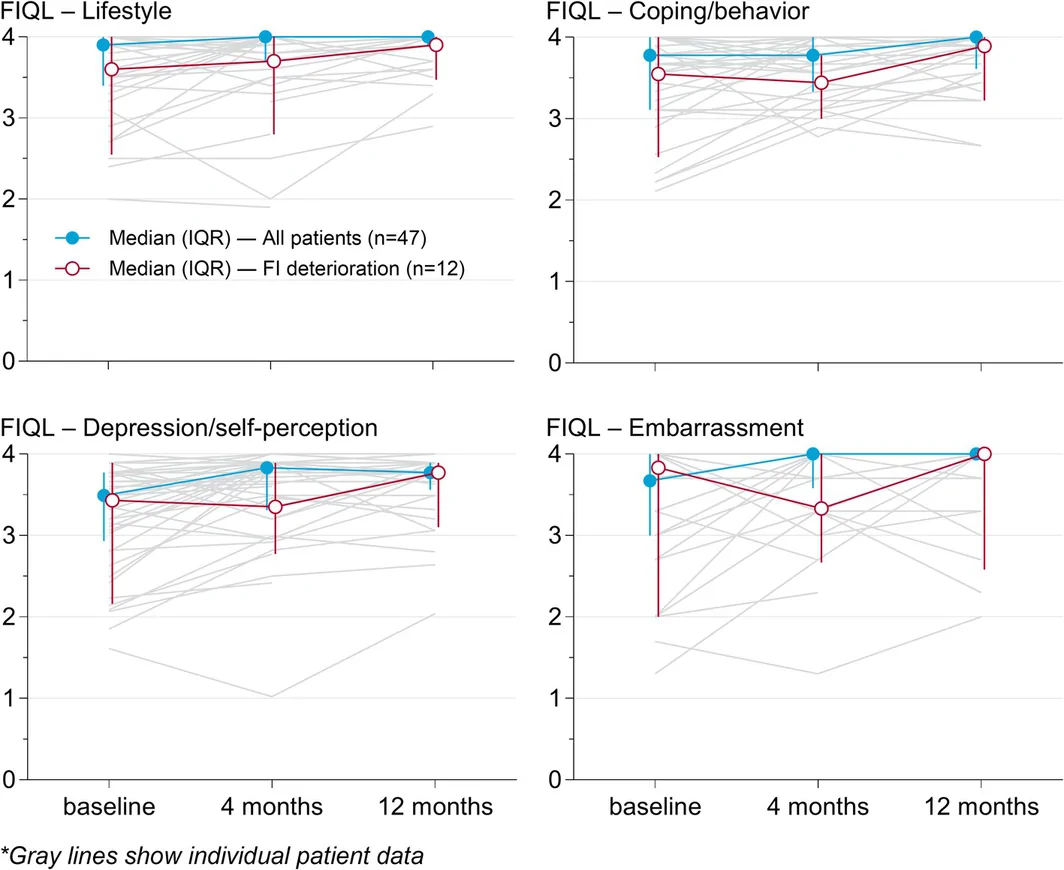
FIQL domain scores during follow‐up.

In addition to quality of life, ProctoPROM and VAS pain scores significantly improved over time. ProctoPROM scores decreased from a median of 3.6 (IQR 1.9–5.4) at baseline to 0.8 (IQR 0.3–2.5) at 4 months (*p* < 0.001) and further declined to 0.2 (IQR 0–1.1) at 12 months (*p* < 0.001) (Figure S1). VAS pain scores also decreased significantly, from a median of 2.0 (IQR 0.5–3.0) at baseline to 0 (IQR 0–1, *p* < 0.001) at follow‐up. Patient satisfaction with surgical outcomes increased over time, with the proportion of patients who reported their symptoms as unacceptable decreasing from 22 (49%) at baseline to five (11%) at 4 months and to just one patient at 12 months (2.5%) (Figure S2). Fistula healing was confirmed in 46/47 (98%) of patients at 4 months using 3D‐EUS, with no recurrences reported during the 1‐year follow‐up period.

## 4 Discussion

This prospective cohort study evaluated the impact of fistulotomy on anorectal pressure profiles using 3D‐HRAM and assessed their relationship with fecal incontinence and quality of life. The primary aim was to determine whether preoperative anorectal pressures could predict postoperative continence deterioration. Our results suggest that, while mean sphincter pressures remain stable after fistulotomy, localized pressure changes at the fistula site may help explain individual continence outcomes.

At 12 months, 26% of patients met the predefined criteria for fecal continence deterioration, a rate comparable to previous reports, although varying definitions across studies complicate direct comparisons [6, 16, 34, 35, 36]. Unlike most studies, which focus on mean continence scores at the cohort level, we analyzed individual continence deterioration, providing a more granular assessment of functional impact.

Although some patients experienced worsening continence, median Wexner score improved from 4 (IQR 1–7) preoperatively to 1 (IQR 0–2) postoperatively, reflecting symptom relief in most patients. This aligns with previous findings, such as Murad‐Regadas et al., who reported similar postoperative Wexner scores and mild incontinence in 31% of cases [18]. However, a key finding of our study is the limited utility of the Wexner score in fistula patients. Preoperative scores were often elevated due to extensive sanitary pad usage and reported incontinence for liquid stool, which likely reflected fistula‐related soiling rather than true sphincter dysfunction. This may explain postoperative score reductions, as fistula healing eliminates soiling symptoms, potentially masking new or persistent continence impairment and overestimating baseline scores. For this reason, we used a composite endpoint incorporating both worsening from baseline and clinically relevant postoperative continence impairment.

Given these limitations, cohort‐level Wexner score changes after fistula surgery may not reliably capture continence deterioration. The recent Songdo Consensus Statement similarly recommends assessing continence on an individual level rather than relying on mean cohort scores, aligning with our approach [34]. The Anal Fistula Core Outcome Set (AFCOS) also advocates for standardized continence evaluation in all fistula studies, both pre‐ and postoperatively [37]. However, regrettably, no specific recommendations are provided regarding the optimal validated scoring system, highlighting the need for a fistula‐specific continence assessment tool that accounts for preoperative fistula‐related soiling.

Our data show that localized pressure loss at the fistula site increased postoperatively, suggesting subtle sphincter impairment that may contribute to continence deterioration in some patients. Clinically, a 1 mmHg lower preoperative FTP was associated with a 9% increase in the odds of continence deterioration (OR 1.09, 95% CI: 1.02–1.16). While the effect size was modest, these findings indicate that preoperative fistula tract pressure measurements may aid in risk stratification.

Interestingly, a greater pressure drop during voluntary squeeze was associated with a lower risk of continence deterioration (OR 0.92), implying that preserved contractility in unaffected sphincter fibers may compensate for local impairment at the fistulotomy site. Thus, even when preoperative FTP are low, continence can be maintained if the remaining sphincter is functionally intact. This supports earlier findings that anatomical defects on EUS do not always correlate with clinical symptoms [34].

Traditional mean sphincter pressures (RPmean, SPmax) were not predictive of continence deterioration, confirming previous studies [38]. Instead, our findings underscore the added value of regional pressure profiling with 3D‐HRAM, particularly fistula tract pressure assessment, as a potentially more sensitive marker of functional risk. However, substantial overlap in pressure values between patients with and without continence deterioration currently limits the clinical utility of fixed pressure thresholds.

Despite these observations, the overall impact of fistulotomy on continence was limited. The healing rate was 98%, and continence deterioration was generally mild. Known risk factors, such as anterior fistulas in women (*n* = 10) and prior fistula surgery (*n* = 20), were not significantly associated with worse continence outcomes in this cohort. However, the small sample size may have limited our ability to detect such associations.

Postoperatively, QoL and daily functioning improved significantly, reinforcing the negative impact of fistulas on patient well‐being and the benefits of successful healing. Interestingly, patients who experienced continence deterioration reported lower FIQL scores at baseline and at 4 months, though these improved by 12 months, suggesting adaptation or improved coping strategies over time. These findings underscore the importance of thorough preoperative counseling to help manage patient expectations.

This study has several strengths. Its prospective design allowed for standardized functional assessment using both 3D‐HRAM and 3D‐EUS, with blinded outcome evaluation by three independent raters to minimize bias. Additionally, all patients were followed for at least 12 months, providing sufficient time to assess fistula healing and functional outcomes.

However, some limitations should be acknowledged. Selection bias may have occurred, as patients with intraoperative concerns about sphincter involvement were excluded, resulting in a cohort skewed toward lower‐risk cases. Postoperative 3D‐HRAM was unavailable in two patients due to refusal or logistical constraints. Operator variability in clinical assessments and surgical procedures may have introduced minor inconsistencies. As discussed, currently available continence scores have limitations in distinguishing fistula‐related symptoms from true fecal incontinence. Furthermore, the follow‐up period of 12 months may not capture very long‐term continence changes that can develop years after fistulotomy. In addition, the regression analyses should be considered exploratory and hypothesis‐generating given the limited number of continence deterioration events. Finally, although this study provides new insights into localized pressure changes, the direct clinical applicability of these findings remains to be established.

In conclusion, 3D‐HRAM allows for regional pressure mapping in patients with perianal fistulas but does not reliably predict continence deterioration following fistulotomy for simple cases. Lower preoperative fistula tract pressures were associated with continence deterioration, but the clinical significance of this finding remains uncertain. Given the overall low risk of severe incontinence, routine preoperative 3D‐HRAM is not warranted for simple fistulas. Future research should focus on refining continence assessment tools and further explore the predictive value of 3D‐HRAM in more complex fistulas, where its clinical utility may be greater.

## Author Contributions

**Pieter van Hagen:** conceptualization, data curation, investigation, validation, methodology, supervision, writing – review and editing. **Lisette Dekker:** conceptualization, data curation, investigation, methodology, writing – review and editing. **Justin Y. van Oostendorp:** conceptualization, investigation, methodology, validation, visualization, writing – review and editing, writing – original draft, formal analysis, data curation, project administration. **Ingrid J. M. Han‐Geurts:** conceptualization, methodology, data curation, validation, investigation, supervision, writing – review and editing. **Ruben Veldkamp:** conceptualization, data curation, methodology, investigation, validation, supervision, writing – review and editing.

## Funding

The authors have nothing to report.

## Disclosure

The authors have nothing to report.

## Supporting information


**Figure S1:** ProctoPROM scores at baseline, 4 and 12 months.

**Figure S2:** Patient‐reported condition acceptability at baseline, 4 and 12 months.

## Data Availability

The data that support the findings of this study are available from the corresponding author upon reasonable request.
